# The clinical utility of bronchoalveolar lavage fluid metagenomic next-generation sequencing in immunocompromised critically ill patients with invasive pulmonary aspergillosis: a multicenter retrospective study

**DOI:** 10.1128/spectrum.00584-25

**Published:** 2025-06-12

**Authors:** Junjie Zhao, Runxi Zhuge, Kai Guo, Jing Tang, Yong Sun, Yi Zhang, Lingmin Yuan, Canhu Qiu, Youqin Yan, Kaiyu Wang, Qin Jiang, Juan Chen, Zhidan Hua, Liyan Qiu, Honglong Fang, Jiancheng Zhuge

**Affiliations:** 1 Zhejiang Chinese Medical University 70571, Hangzhou, Zhejiang, China; 2 Shanghai Medical College, Fudan University 58305, Shanghai, China; 3 Department of Critical Care Medicine, QuZhou KeCheng People’s Hospital, Quzhou, Zhejiang, China; 4 Department of Critical Care Medicine, Longyou County People’s Hospital, Quzhou, Zhejiang, China; 5 Department of Critical Care Medicine, Jiangshan People’s Hospital, Quzhou, Zhejiang, China; 6 Department of Critical Care Medicine, People’s Hospital of Changshan County, Quzhou, Zhejiang, China; 7 Department of Critical Care Medicine, The Quzhou Affiliated Hospital of Wenzhou Medical University, Quzhou People’s Hospital, Quzhou, Zhejiang, China; 8 Nursing Department, Sir Run Run Shaw Hospital, Zhejiang University School of Medicine, Hangzhou, China; 9 Department of Clinical Laboratory, The Quzhou Affiliated Hospital of Wenzhou Medical University, Quzhou People’s Hospital, Quzhou, Zhejiang, China; 10 Plmonary and Critical Care Medicine, The Quzhou Affiliated Hospital of Wenzhou Medical University, Quzhou People’s Hospital, Quzhou, Zhejiang, China; 11 Quzhou TCM Hospital at the Junction of Four Provinces Affiliated to Zhejiang Chinese Medical University, Quzhou, Zhejiang, China; University of Maryland School of Medicine, Baltimore, Maryland, USA

**Keywords:** etiology, immunocompromised patients, bronchoalveolar lavage fluid, invasive pulmonary aspergillosis, metagenomic next-generation sequencing

## Abstract

The clinical utility of metagenomic next-generation sequencing (mNGS) in immunocompromised critically ill patients with invasive pulmonary aspergillosis (IPA) remains poorly studied. Given the diagnostic challenges and high mortality associated with IPA in this population, further research on the use of mNGS for early diagnosis and targeted therapy is urgently needed. This multicenter, retrospective, observational study enrolled immunocompromised patients admitted to the intensive care units of six tertiary hospitals in China from April 2021 to November 2024. Comprehensive clinical data were systematically collected, including demographic information, underlying conditions, and detailed records of specimen types and microbiological examination methods. The primary objective was to evaluate the diagnostic and prognostic values of mNGS in comparison to conventional microbiological tests (CMTs) for IPA in this high-risk population. Kappa analysis results indicated a significant agreement between the results of mNGS and CMTs in both groups (Kappa value = 0.638, *P* < 0.001). The receiver operating curve demonstrated that mNGS exhibited comparable sensitivity (94.03% vs. 95.52%) and higher specificity (96.20% vs. 78.85%), as well as a higher area under the curve (AUC) (0.951 vs. 0.872) in diagnosing IPA compared to CMTs. Moreover, mNGS was significantly superior to other single methods, including cultures (AUC: 0.620, sensitivity: 27.88%, specificity: 96.15%), galactomannan test (AUC: 0.711, sensitivity: 53.73%, specificity: 88.46%), and PCR (AUC: 0.770, sensitivity: 62.69%, specificity: 91.35%). The clinical application of mNGS-guided antibiotic adjustments significantly decreased the 28-day mortality rate (46.51% vs. 66.67%, *P* < 0.05). mNGS is a feasible and highly sensitive diagnostic tool for detecting *Aspergillus* infections in immunocompromised critically ill patients compared to CMTs and other single conventional methods. It also performs well in identifying mixed infections, facilitating appropriate antibiotic regimen adjustments and improving patient prognosis.

**IMPORTANCE:**

mNGS demonstrated significantly higher specificity and area under the curve for diagnosing IPA in immunocompromised critically ill patients compared to CMTs. mNGS showed superior diagnostic performance over single methods, such as cultures, galactomannan test, and PCR, with higher sensitivity and specificity for *Aspergillus* detection. The use of mNGS-guided antibiotic adjustments led to a significant reduction in 28-day mortality (46.51% vs. 66.67%) among immunocompromised patients. mNGS demonstrated utility in identifying mixed infections, supporting targeted therapy and better patient outcomes. The application of mNGS in diagnosing IPA and guiding treatment in ICU patients helped optimize antibiotic regimens, ultimately improving clinical prognosis.

## INTRODUCTION

Recent studies have highlighted that immunocompromised patients constitute a substantial proportion of intensive care unit (ICU) admissions, with approximately one-third of ICU patients presenting at least one risk factor for immunosuppression (1, 2). Immunosuppression, whether due to underlying diseases or therapeutic interventions, significantly increases the risk of pulmonary fungal infections. Among these, invasive pulmonary aspergillosis (IPA) is the most common and often the most fatal. The pathophysiological mechanisms underlying IPA in immunocompromised individuals are complex and multifactorial, involving both impaired immune surveillance and disruption of pulmonary defenses. Given the high morbidity and mortality associated with IPA, early diagnosis and appropriate treatment are crucial for improving patient outcomes (3).

IPA predominantly affects severely immunocompromised hosts, particularly neutropenic patients. In contrast, the isolation of *Aspergillus* species (spp.) in immunocompetent individuals is generally considered colonization rather than infection (4, 5). However, with the widespread use of antibiotics, corticosteroids, and immunosuppressive therapies, the incidence of IPA has significantly increased among immunocompromised patients in recent years. In these patients, the diagnosis of IPA is often delayed due to nonspecific clinical manifestations, leading to delayed initiation of antifungal treatment and, consequently, higher mortality rates (6, 7).

Histopathology is commonly regarded as the “gold standard” for diagnosing *Aspergillus* infections; however, challenges in obtaining a sufficient biopsy sample from the infected site often result in a high rate of false-negative results (8). Although fungal culture also remains one of the standard methods for diagnosing *Aspergillus* infections, it is time-consuming and has relatively low sensitivity, often leading to delayed diagnosis. Both (1,3)-β-D glucan (G) and galactomannan (GM) assays, though widely used, have limitations (9, 10). These limitations include the delayed peak concentrations of biomarkers and potential interference from medications, such as antibiotics and antifungal agents, which can reduce their diagnostic accuracy, particularly in the early stages of infection. While polymerase chain reaction (PCR)-based methods are more rapid, sensitive, and specific, they require specific primers and are not routinely performed in many hospitals. Furthermore, the nonspecific radiological features of pulmonary involvement significantly limit the diagnostic value of chest computed tomography (CT).

Metagenomic next-generation sequencing (mNGS) directly performs high-throughput sequencing of nucleic acids in clinical samples, enabling comprehensive comparison with extensive reference databases to identify and quantify a wide range of microbial species. This cutting-edge technology has demonstrated significant potential for the early diagnosis of IPA in recent years (7, 9, 11, 12). However, current research on the application of mNGS in immunocompromised critically ill patients with IPA remains limited. Additionally, nucleic acid enrichment from fungal species is less efficient compared to bacteria or viruses due to challenges in the lysis of fungal polysaccharide cell walls (13). Therefore, this study aims to further validate the clinical value of mNGS for the early diagnosis of IPA in this population and to explore its impact on prognosis.

## MATERIALS AND METHODS

### Patients

This multicenter, retrospective, observational study was approved by the local ethics committee and conducted in accordance with the Declaration of Helsinki. All research was performed following relevant guidelines/regulations. Informed consent was obtained from all participants and/or their legal guardians. From April 2021 to November 2024, 186 immunocompromised patients suspected of having pneumonia in the ICU were reviewed at six hospitals in China.

The inclusion criteria were as follows: (i) immunocompromised patients, including hematological malignancy (active or in remission for less than 5 years), solid organ transplantation or hematopoietic stem cell transplantation, neutropenia or chemotherapy for solid tumors in the past 3 months, use of immunosuppressants, biological immunomodulators, and antirheumatic drugs (e.g., methotrexate, cyclophosphamide, and cyclosporin), daily intake of more than 20 mg of glucocorticoids for more than 14 days (or a cumulative dose of 700 mg prednisolone or equivalent doses of other corticosteroids), and immunocompromised status due to hereditary or congenital factors (14); (ii) undergoing bronchoalveolar lavage, with culture, GM or G test galactomannan and mNGS results; and (iii) detection results for other pathogens, including but not limited to bacterial culture, acid-fast staining, and PCR test for *Pneumocystis jirovecii*, *Mycobacterium tuberculosis*, and cytomegalovirus (CMV). The exclusion criteria were as follows: (i) incomplete clinical data; (ii) conventional microbiological tests (CMTs) and mNGS not tested simultaneously; and (iii) age < 18 years old.

**Fig 1:**
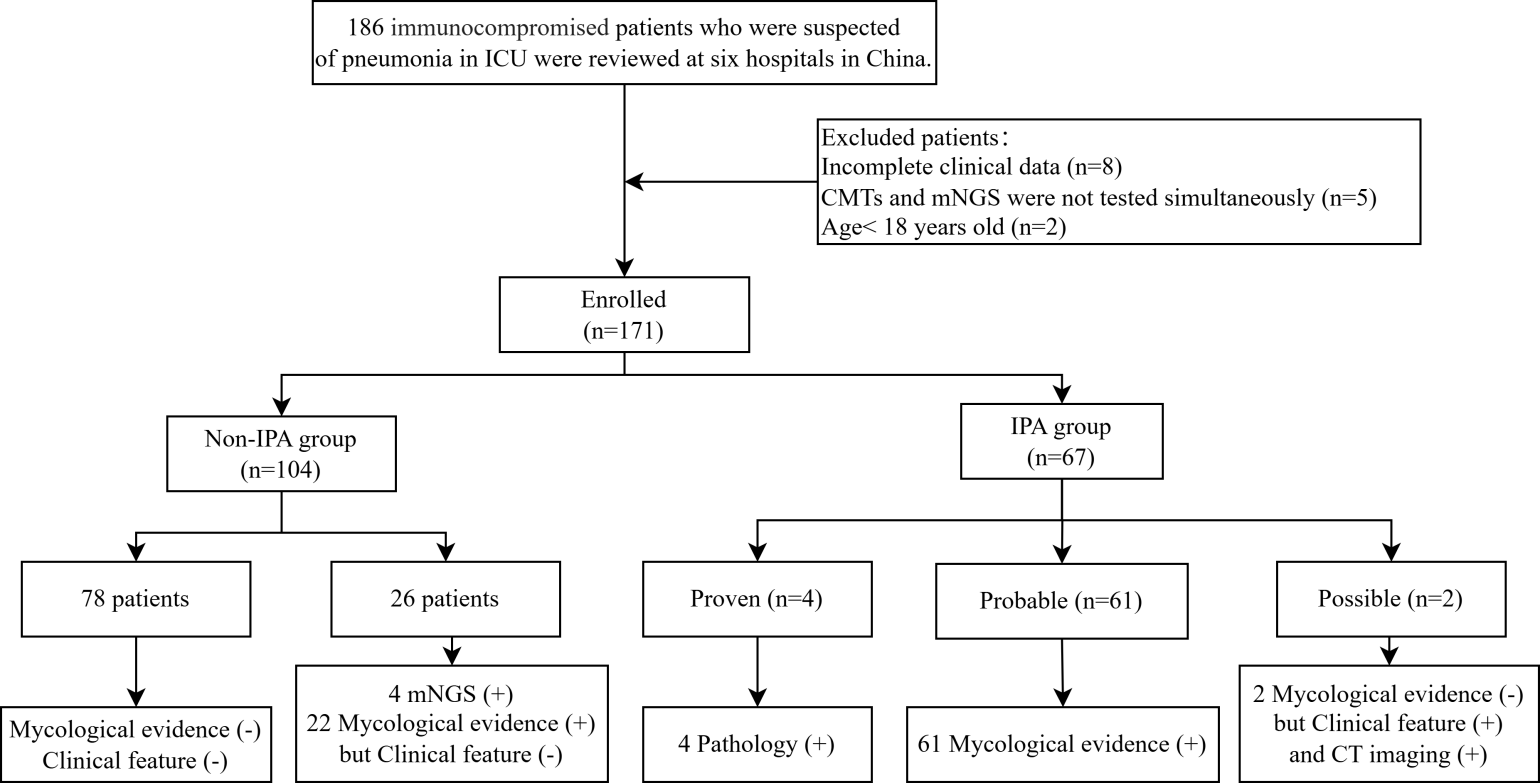
Study flow diagram. Abbreviations: mNGS, metagenomic next-generation sequencing; CMTs, conventional microbiological tests; IPA, invasive pulmonary aspergillosis; CT, computed tomography.

According to the inclusion and exclusion criteria, a total of 171 patients were finally included in our study. According to the diagnostic criteria for IPA, patients were classified into two groups: the IPA and non-IPA groups (Fig. 1).

### Criteria for the diagnosis of IPA

For proven IPA: histopathology, cytopathology, or direct microscopy of specimens obtained by needle aspiration or biopsy revealed hyphae and accompanied by evidence of associated tissue damage. For probable IPA, the presence of at least one host factor, clinical feature, and mycologic evidence was required according to the 2020 criteria from the European Organization for Research and Treatment of Cancer/Invasive Fungal Infections Cooperative Group (15). When any of the following thresholds is met, it will be used as mycological evidence: (i) culture and/or histopathological examination positive for *Aspergillus*; (ii) the result of GM test was considered positive when (at least one): single serum or plasma ≥ 1.0, BALF ≥ 1.0, single serum or plasma ≥ 0.7, and BALF ≥ 0.8; (iii) at least two positive PCR tests in plasma, serum, whole blood, or BAL fluid or at least one positive PCR test in both plasma/serum/whole blood and BAL fluid; and (iv) *Aspergillus* spp. recovered by culture from sputum, BALF, bronchial brush, or aspirate. For possible IPA: meet the criteria of a host factor or a clinical feature or a positive CT imaging result, but for which mycological evidence has not been found. Patients with positive mycological cultures or amplification of fungal DNA without signs of infection were classified as *Aspergillus* colonization (15). Therefore, cases were considered as colonization when *Aspergillus* was identified but without a final diagnosis of *Aspergillus* infection.

Patients who were clinically diagnosed with IPA were classified into the IPA group. For immunocompromised patients with no clinical features and no mycological evidence, they were classified as the non-IPA group in this study. The final diagnosis was established based on the patients’ host factors, clinical characteristics, and microbiological test results. The final diagnosis was established. These patients were suspected of IPA, with a total of 171 patients included (67 with IPA and 104 without IPA).

### Conventional microbiological tests

Pathogen identification was performed using BALF, blood, sputum, and other respiratory tract samples through CMTs. These included bacterial cultures on blood, chocolate, and MacConkey agar plates, as well as fungal cultures on Sabouraud agar plates. Fungi were additionally detected using the GM test, PCR, and fungal culture. Cryptococcus antigens were identified through serum antigen detection, while *Mycobacterium* spp. was tested using T-spot and GeneXpert. Viruses were identified via PCR. Smear microscopy was conducted for fungi using fluorescent and KOH stains and for tuberculosis using acid-fast staining.

### mNGS procedure

#### DNA/RNA extraction

BALF samples were centrifuged at 12,075 ×*g* and 4°C for 5 min. For each sample, 500 µL of the supernatant was used to extract genomic DNA using the PathoXtract WYXM03202S Universal Pathogen Enrichment Extraction Kit (WillingMed, Beijing, China), and RNA was extracted using the PathoXtract Virus DNA/RNA Isolation Kit (WYXM03009S, WillingMed Corp, Beijing, China) according to the manufacturer’s instructions.

#### Construction of DNA/RNA libraries and sequencing

To detect DNA pathogens, DNA libraries were constructed using the Illumina DNA Prep (M) Tagmentation Kit (20018705; Illumina, San Diego, USA). For co-detection of DNA and RNA pathogens, DNA and RNA were first combined, and then RNA was reverse-transcribed into complementary DNA (cDNA) using the SuperScript Double-Stranded cDNA Synthesis Kit (11917020, Invitrogen). Library quality was assessed using both a Qubit fluorescence quantification analyzer (Thermo Fisher) and an Agilent 2100 Bioanalyzer (Agilent Technologies). Sequencing was performed on an Illumina NextSeq 550Dx sequencer (San Diego, USA), achieving a minimum of 20 million reads per sample.

#### Bioinformatic analysis

High-quality sequencing data were obtained by removing low-quality reads, adaptor-contaminated reads, reads with excessive low-complexity regions, and reads with insufficient length using Trimmomatic v0.40 (16). Human-derived sequences were subsequently filtered out by aligning reads to the human reference genome using Bowtie2 v2.4.3 (17). The remaining sequences were aligned to four databases downloaded from National Center for Biotechnology Information, including bacteria, fungi, viruses, and parasites, for classification after removal of human host sequences with Kraken2 v2.1.0 (18).

### Criteria for positive mNGS results

For identification of the pathogens, a reads per 10 million (RPTM) value, which is defined as detected number of pathogen-specific reads per 10 million, was used. The RPTM ratio metric, defined as RPTMSample/RPTMNTC, is set to one in NTC samples when the microbial taxonomic value of a given species or genus is less than 1 (19). For viruses, the criterion was a reads per million ratio of ≥3.0; for bacteria and fungi, the criterion was an RPTM ratio of ≥20.0 (20); for special pathogens, including *Cryptococcus*, *Mycobacterium*,* Nontuberculous mycobacteria*, etc., with RPTM ≥ 1 was identified as positive and similar pathogens (19, 21).

To exclude pathogens suspected of being colonizers, we compared the detected microbial species with an in-house background microbial database. This database contains information on common bacterial species that are typically found as part of the normal microbiota in our patient population. Any bacterial species that matched the profile of known colonizers in the database were excluded from the analysis to focus on potential pathogens (22).

The consistency of the results between mNGS and CMTs for clinical diagnosis was comprehensively assessed by two experienced clinicians with over 10 years of ICU experience (Fang HL and Zhuge JC), leading to a final diagnosis based on the comprehensive results. The diagnosis took into account the patients’ clinical characteristics, results of CMTs, results of mNGS, laboratory data, and other relevant factors. Additionally, chest CT and other imaging modalities are also employed to assist in the overall diagnostic process.

### Concordance of the results of mNGS and CMTs

To evaluate the diagnostic consistency between mNGS and CMTs for detecting *Aspergillus* spp., we employed the Kappa consistency test. Additionally, to highlight the differences between mNGS and CMTs in detecting *Aspergillus* and other pathogenic microorganisms, we defined the following criteria and used a composite pie chart to visually represent the results:

1. **Complete match**: Both mNGS and CMTs detected *Aspergillus* infection, and the identification of other pathogenic microorganisms was consistent between the two methods.
2. **Partial match**: Both mNGS and CMTs detected *Aspergillus* infection, but mNGS or CMTs identified different or additional pathogenic microorganisms.

### Evaluation of the clinical impact of mNGS or CMT results

**Fig 2:**
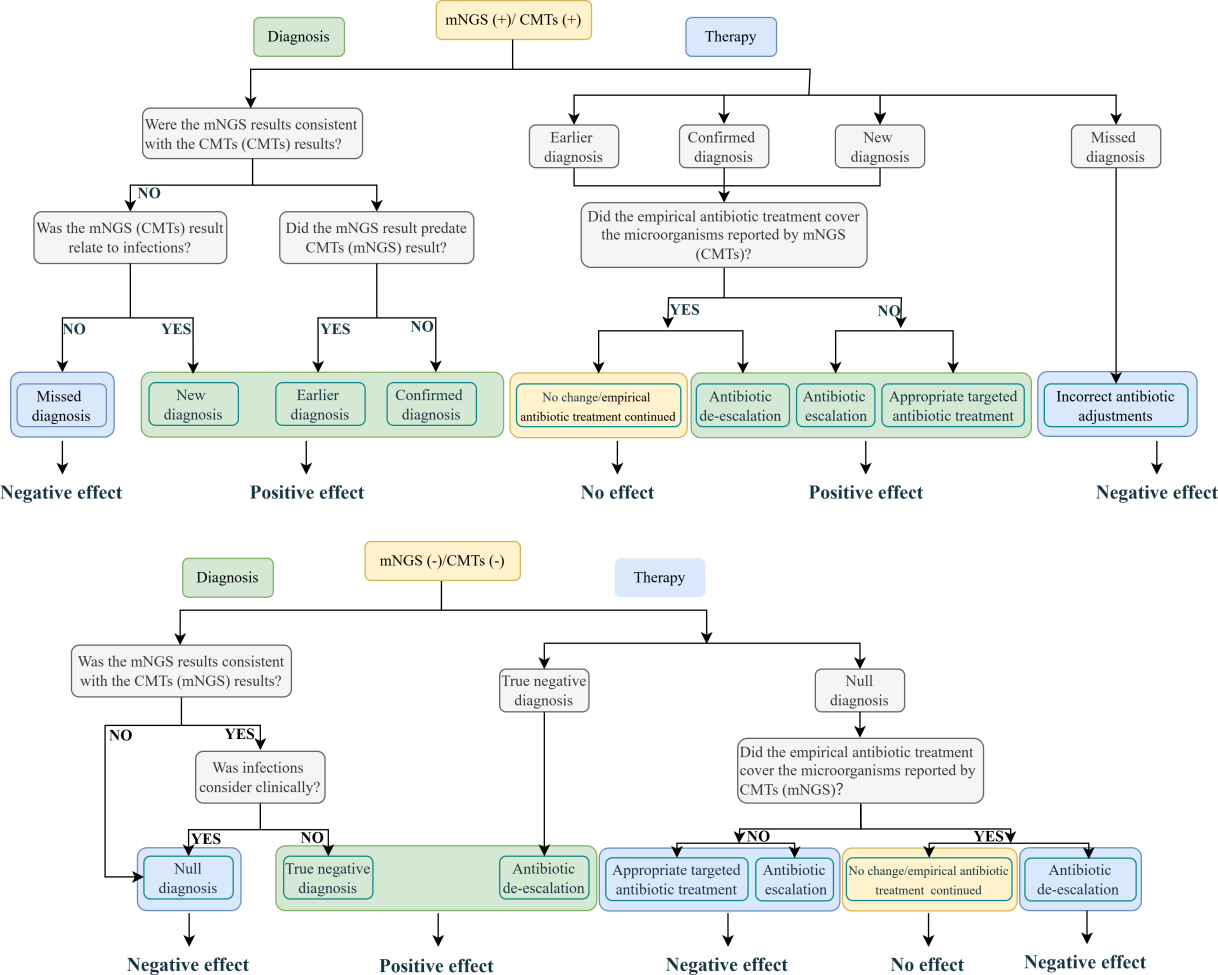
Evaluation procedures for the clinical impact of mNGS (CMT) results.

To further evaluate the clinical impact of mNGS or CMT results, we categorized the impact as positive, negative, or no effect (Fig. 2). Clinical impact types were categorized into therapy- and diagnosis-related impacts based on previous studies (23). Positive effects included cases where mNGS or CMT results led to antibiotic escalation, de-escalation (including discontinuation), or appropriate targeted treatment (for fungi, viruses, or tuberculosis). Negative effect referred to cases where mNGS or CMT results caused a misdiagnosis, leading to incorrect antibiotic adjustments. No effect was defined as cases where mNGS or CMT results had no significant clinical impact, and empirical antibiotic treatment was continued.

### Statistical analysis

Data were statistically analyzed using SPSS (version 26; IBM Corp., Armonk, NY, USA) and R software (version 4.4.1; R Foundation for Statistical Computing, Vienna, Austria). Figures were created using GraphPad Prism (version 10; GraphPad Software, San Diego, CA, USA) and OriginPro (version 2024b; OriginLab Corp., Northampton, MA, USA). Continuous variables were tested for normality using the Kolmogorov-Smirnov test and expressed as median and interquartile range (IQR) or as (mean ± SD) depending on the distribution. The receiver operating characteristic (ROC) curves were plotted to analyze the diagnostic value of mNGS or CMTs in immunocompromised critically ill patients with IPA. Data comparisons were performed using an independent samples *t*-test or the Mann-Whitney *U* test. Categorical variables were compared using the *χ* test. The Kappa consistency test was conducted to determine whether the diagnostic results of the two methods were consistent. In this study, *P* < 0.05 (two-tailed test) was considered statistically significant.

## RESULTS

### Clinical characteristics of patients

From April 2021 to November 2024, 186 immunocompromised patients suspected of having pneumonia were admitted to ICUs at six hospitals in China. After excluding eight patients with incomplete clinical data, five patients who did not undergo simultaneous CMTs and mNGS testing, and two patients under the age of 18, 171 patients were included in the study. Based on comprehensive clinical diagnoses, patients were divided into the IPA (*n* = 67) and non-IPA groups (*n* = 104). In the IPA group, four patients were diagnosed with proven IPA, 61 patients with probable IPA, and two patients with possible IPA (Fig. 1).

The demographic and clinical characteristics of both groups are summarized in Table 1. The median age of patients in the IPA group was significantly higher than that in the non-IPA group, that is, (69 [61–75]) vs. 66 [55–75], *P* = 0.035). The most common cause of immunocompromised conditions in both groups was prolonged corticosteroid or immunosuppressants (49.25% vs 37.50%, *P* = 0.130), and the proportion of neutropenic patients in the IPA group was significantly higher (41.79% vs. 11.54%, *P* = 0.000). Clinical laboratory tests revealed that the white blood count (WBC) and neutrophil counts in the IPA group were significantly lower than those in the non-IPA group (all *P* < 0.05).

**TABLE 1:** Clinical characteristics of patients^*a*^

	IPA group (*n* = 67)	Non-IPA group (*n* = 104)	*P* value
Age, years, median (IQR)	69 (61–75)	66 (55–75)	0.035
Gender (*n*, %)			0.587
Male	37 (55.22%)	53 (50.96%)	
Female	30 (44.78%)	51 (49.04%)	
Immunocompromised conditions (*n*, %)			
Hematologicc malignancy (without neutropenia)	6 (8.96%)	34 (32.69%)	<0.001
Active solid tumor	10 (14.93%)	26 (25.00%)	0.185
Neutropenia	28 (41.79%)	12 (11.54%)	<0.001
Prolonged corticosteroid or immunosuppressants	33 (49.25%)	39 (37.50%)	0.130
Clinical laboratory tests, median (IQR)			
WBC, ×10^9^/L	6.30 (3.00–12.90)	8.80 (5.00–13.25)	0.024
PCT, ng/mL	1.90 (0.27–14.20)	3.18 (0.55–14.21)	0.360
CRP, mg/L	78.92 (22.43–162.20)	87.99 (35.43–173.33)	0.360
Platelets, ×10^9^/L	129.00 (80.00–194.00)	106.50 (71.25–172.00)	0.098
Hemoglobin, g/L	110.00 (92.00–119.00)	107.50 (91.25–121.25)	0.727
Neutrophil count, ×10^9^/L	4.84 (1.81–11.89)	7.28 (3.96–11.21)	0.027
Bilirubin, μmol/L	6.23 (4.30–11.20)	6.32 (3.98–10.63)	0.972
Creatinine, μmol/L	62.50 (50.90–88.40)	69.80 (57.35–118.45)	0.104
D-dimer, mg/L	4.04 (2.06–9.36)	2.41 (1.23–6.20)	0.990
Lactic acid, mmol/L	1.90 (1.40–2.70)	1.81 (1.40–2.60)	0.759
Disease severity			
APACHE-II, median (IQR)	16 (12–22)	15 (10–21)	0.145
CURB-65, median (IQR)	2 (1–3)	2 (1–3)	0.535
Score ≥ 3 (*n*, %)	22 (32.84%)	33 (31.73%)	
Score < 3 (*n*, %)	45 (67.16%)	71 (68.27%)	
SOFA, median (IQR)	12 (9–15)	13 (10–16)	0.863
ICU treatment			
MV (*n*, %)	60 (89.55%)	89 (85.58%)	0.312
Length of MV days, median (IQR)	7 (2–11)	7 (3–16)	0.771
Vasopressor (*n*, %)	53 (79.10%)	77 (74.04%)	0.450
Length of ICU stay (day)	9 (7–12)	9 (5–15)	0.771
ICU mortality (*n*, %)	30 (44.78%)	20 (19.23%)	<0.001
28-Day mortality (*n*, %)	36 (53.73%)	34 (32.69%)	0.006

^*a*^ Abbreviations: WBC: white blood count; PCT: procalcitonin; CRP: c-reactive protein; APACHE-II: Acute Physiology and Chronic Health Evaluation II; MV: mechanical ventilation; IQR: interquartile range; ICU: intensive care unit; CURB-65: confusion, urea, respiratory rate, blood pressure, age 65 and older.

There were neither significant differences in disease severity scores between the two groups nor in the use of mechanical ventilation (MV), and vasopressors also showed no significant differences (all *P* > 0.05). However, ICU mortality (44.78% vs. 19.23%, *P* = 0.000) and 28-day mortality (53.73% vs. 32.69%, *P* = 0.006) were significantly higher in the IPA group compared to the non-IPA group.

### Difference between the two groups in detecting pathogenic microorganisms by using mNGS or CMTs

**Fig 3:**
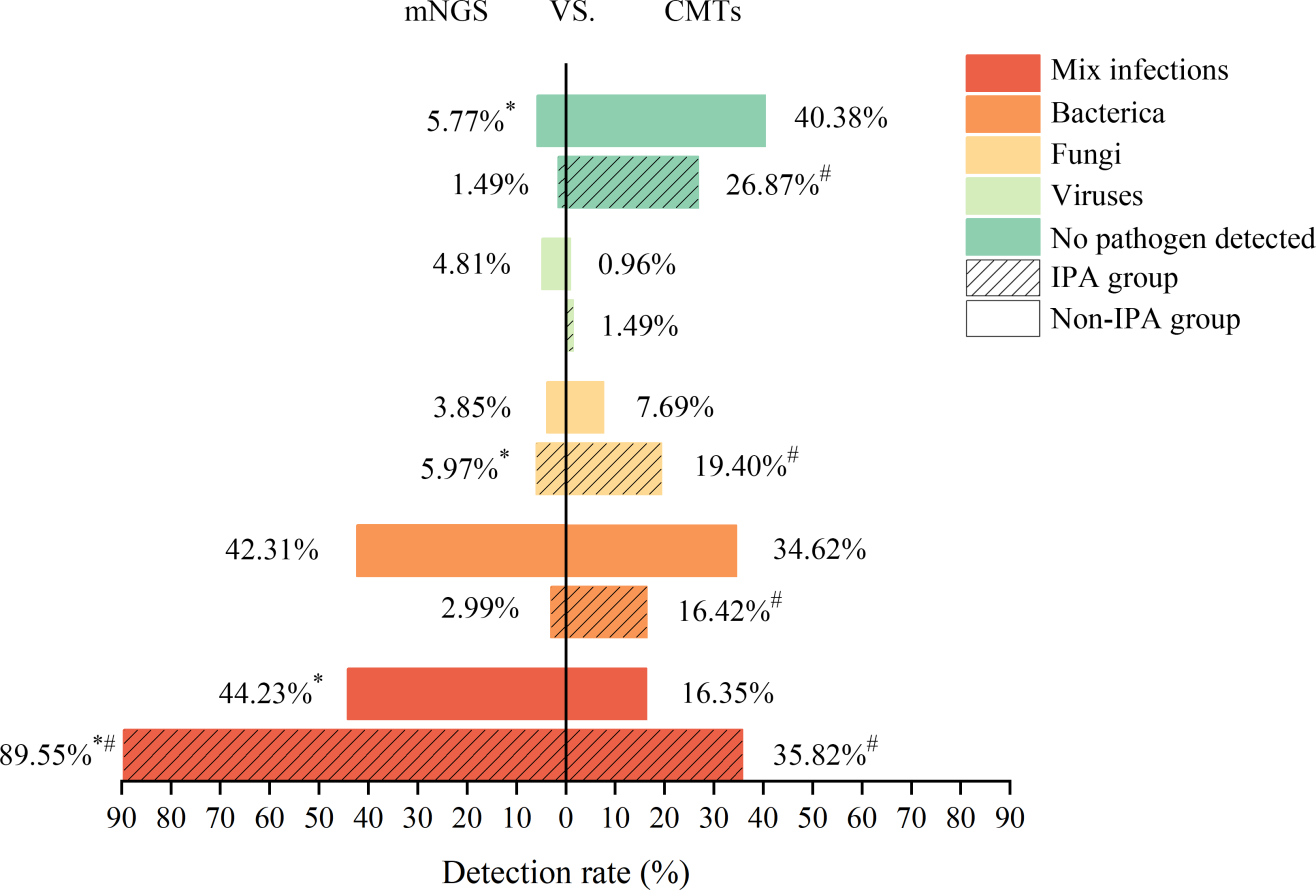
Categorization of microorganism infections in patients based on mNGS or CMT results. *P* < 0.05, mNGS result compared to CMT result. **^#^***P* < 0.05, comparison of mNGS or CMT results between the IPA and non-IPA groups.

Our study results showed that the detection rate of mixed infections using mNGS was significantly higher than with CMTs in both groups (all *P* < 0.05). Additionally, the rate of no pathogens detected was significantly lower with mNGS compared to CMTs (all *P* < 0.05, Fig. 3). In the IPA group, although only fungal infections reached statistical significance (5.97% vs. 19.40%, *P* < 0.05) compared to CMTs, the detection rate of single-type infections in the IPA group was also lower with mNGS than with CMTs. Further analysis indicated that both mNGS and CMTs had a significantly higher detection rate of mixed infections in the IPA group compared to the non-IPA group (all *P* < 0.05). The detection rate of single bacterial infections by CMTs was significantly lower in the IPA group than in the non-IPA group (16.42% vs. 34.62%, *P* < 0.05), while the reverse trend was observed for single fungal infections (19.40% vs. 7.69%, *P* < 0.05). Additionally, the rate of no pathogens detected by CMTs was significantly lower in the IPA group than in the non-IPA group (26.87% vs. 40.38%, *P* < 0.05).

**Fig 4:**
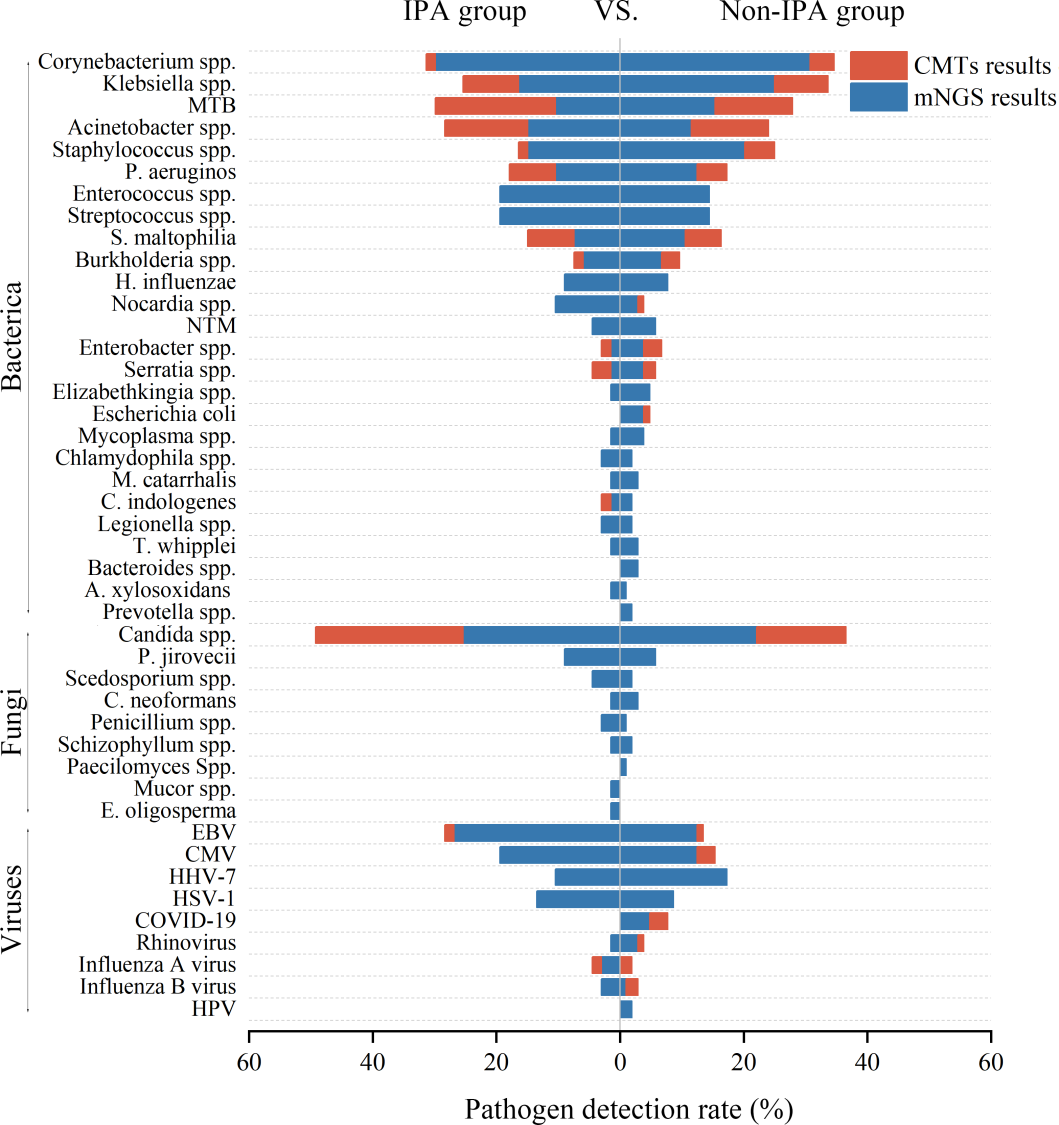
Distribution of the diagnostic rate of microorganisms by mNGS or CMTs. Note: Fig. 4 presents the detection rates of pathogens identified by mNGS or CMTs. These rates only reflect the pathogens detected in the patients and do not necessarily correspond to clinically confirmed infections, as some may represent colonization or sample contamination (the distribution of *Aspergillus* spp. is not depicted in this figure).

Figure 4 shows the differences in pathogen distributions between the two groups (without *Aspergillus* spp.*)*. Compared to CMTs, mNGS demonstrated a significant advantage in pathogen detection rates in both groups. In the IPA group, the most common bacterial infections were caused by *Corynebacterium* spp., *Mycobacterium tuberculosis* (MTB), and *Acinetobacter* spp. In contrast, the non-IPA group exhibited a different pattern, with the most common bacterial infections being *Corynebacterium* spp., MTB, and *Staphylococcus* spp.

In terms of fungal infections, *Candida* spp. and *Pneumocystis jirovecii* were the most common in both groups. Notably, except for *Candida* spp., CMTs failed to detect any other types of fungal pathogens.

Epstein-Barr virus (EBV) and CMV were the most commonly detected viral pathogens in both groups. In the IPA group, the detection rate of herpes simplex virus-1 was higher, while the non-IPA group had a higher prevalence of human herpesvirus 7 infections.

### Concordance of the result of mNGS and CMTs

The Kappa consistency test was performed to evaluate the diagnostic consistency between mNGS and CMTs in both groups. The Kappa analysis results indicated a significant agreement between mNGS and CMTs in both groups (Kappa value = 0.638, *P* = 0.000) (Table 2).

**TABLE 2:** Kappa analysis of concordance between mNGS and CMTs for detecting *Aspergillus*^*a*^

mNGS	CMTs		Total
	Positive (+)	Negative (−)	
Positive (+)	61	6	67
Negative (−)	25	79	104
Total	86	85	171

^*a*^ *P* = 0.000, Kappa value = 0.638.

**Fig 5:**
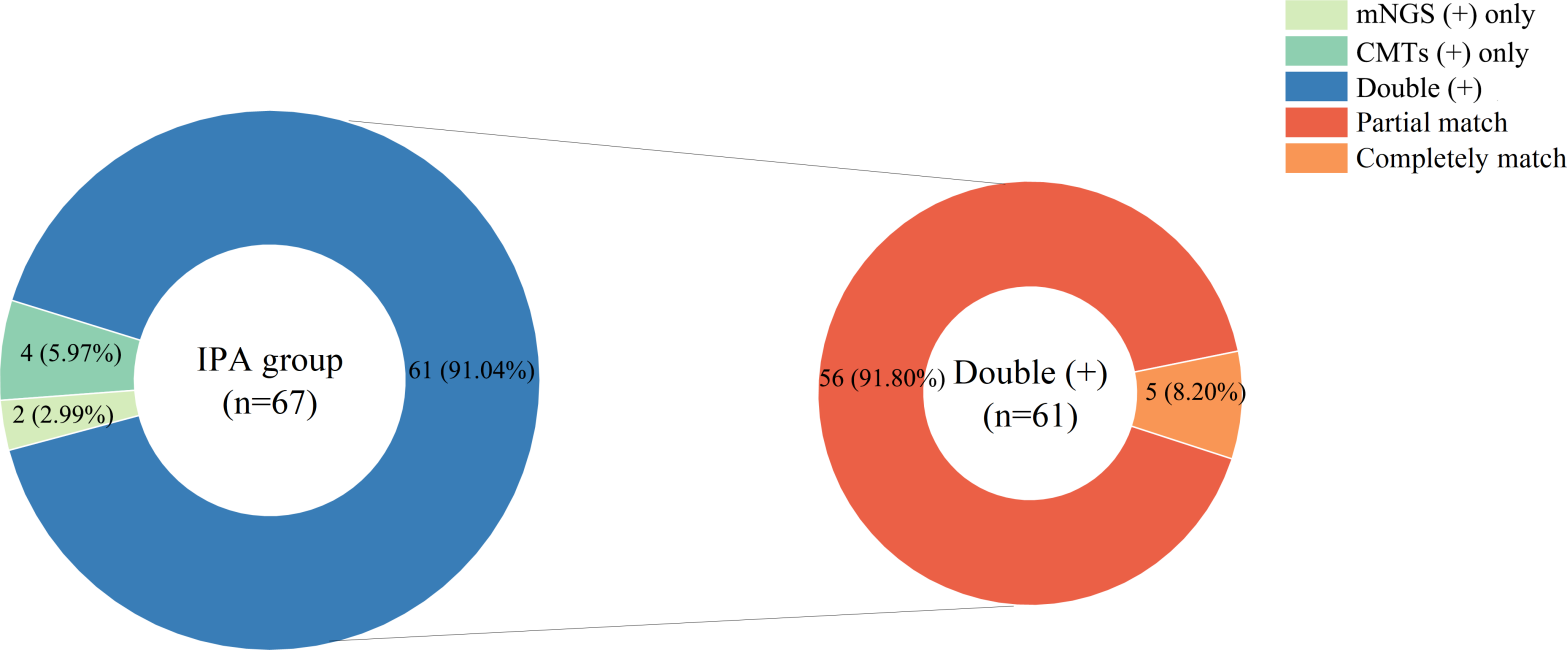
Microorganism identification consistency between mNGS and CMTs in the IPA group.

In the IPA group, 61 patients (91.04%) had positive results for both mNGS and CMTs, while none (0%) had both tests negative. Additionally, two patients (2.99%) had positive mNGS results only, and four patients (5.97%) had positive CMT results only. Furthermore, among the 61 patients who tested positive with both methods, only five patients (8.20%) were completely matched, and 56 patients (91.80%) were partially matched (Fig. 5).

### Clinical impact of mNGS or CMTs result in IPA patients

**Fig 6:**
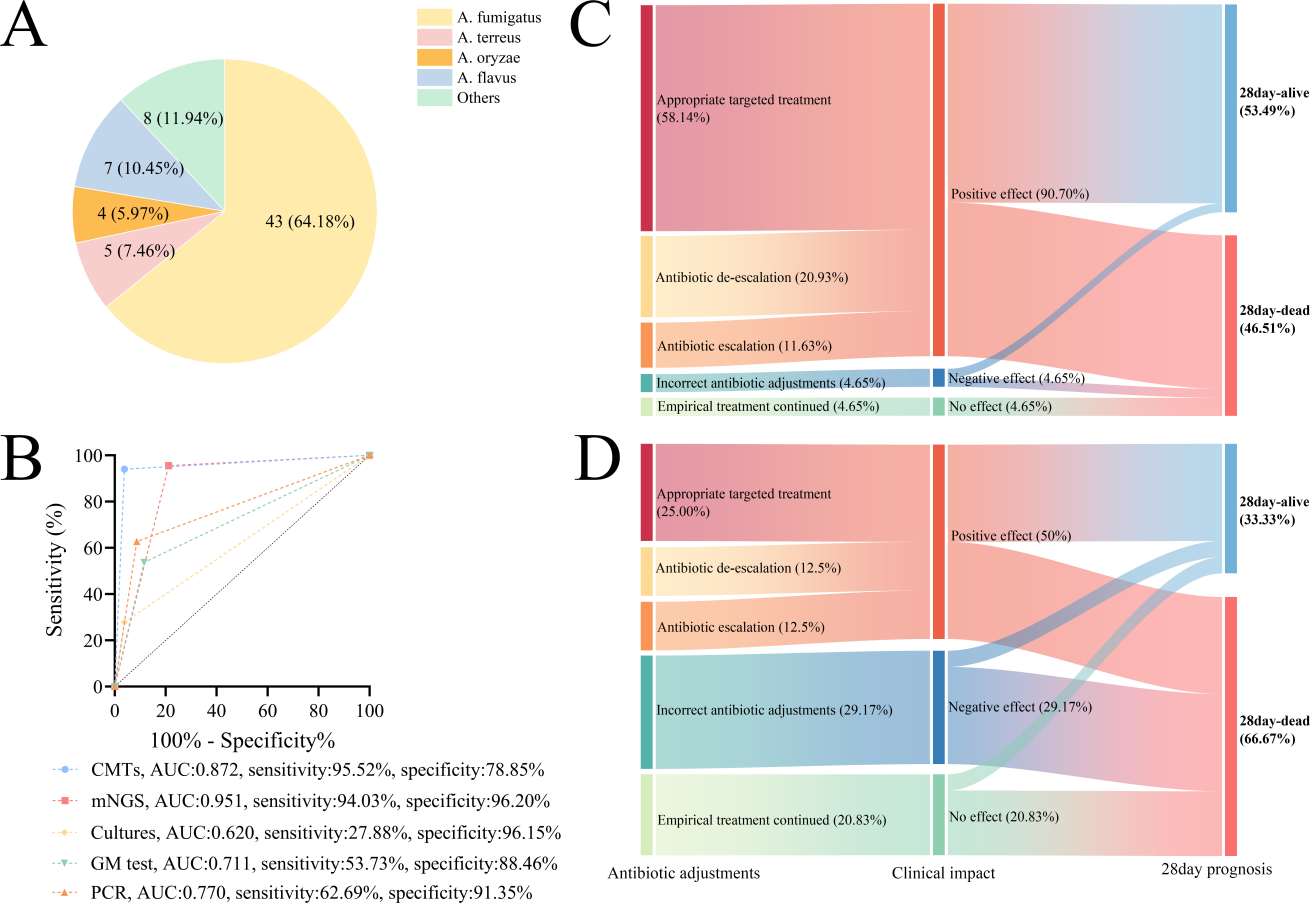
Clinical impact of mNGS or CMT result on IPA patients. (**A**) Distribution of *Aspergillus* spp. in IPA patients. (**B**) ROC curves for mNGS and CMTs in diagnosing IPA. (**C**) Sankey diagram of the clinical impact of antibiotic adjustments guided by mNGS or (**D**) CMTs in IPA patients. Note: in Fig. 6A, others include *Aspergillus sydowii*, *Aspergillus clavatus*, *Aspergillus versicolor*, *Aspergillus chevalieri*, *Aspergillus niger*, *Aspergillus nidulans*, *Aspergillus ruber*, and *Aspergillus tubingensis*.

To further evaluate the clinical impact of mNGS and CMTs on IPA patients, we conducted an in-depth analysis of this population. Among the 67 IPA patients, the most common *Aspergillus* spp. was *A. fumigatus* (*n* = 43, 64.18%), *A. flavus* (*n* = 7, 10.45%), and *A. terreus* (*n* = 5, 7.46%) (Fig. 6A). The ROC curves showed that although both mNGS and CMTs exhibited high sensitivity in diagnosing IPA (94.03% vs. 95.52%), mNGS demonstrated higher specificity and AUC compared to CMTs (AUC: 0.951 vs. 0.872, specificity: 96.20% vs. 78.85%). mNGS was also significantly superior to any other single method: cultures (AUC: 0.620, sensitivity: 27.88%, specificity: 96.15%), GM test (AUC: 0.711, sensitivity: 53.73%, specificity: 88.46%), and PCR (AUC: 0.770, sensitivity: 62.69%, specificity: 91.35%) (Fig. 6B).

Furthermore, we evaluated the clinical impact of antibiotic regimen adjustments based on the results of the two methods on the prognosis in IPA patients. The results showed that antibiotic adjustments guided by mNGS results had a significantly higher positive impact than CMTs (90.70% vs. 50.0%, *P* < 0.05), while the negative impact and no impact rates were both significantly lower with mNGS than with CMTs (all *P* < 0.05). The clinical impact of these methods significantly also affected the 28-day mortality rate of patients (Fig. 6C and D). Interestingly, due to the significantly higher positive impact, the 28-day mortality rate under mNGS-guided adjustments was notably lower than CMTs, demonstrating a better prognosis.

## DISCUSSION

Opportunistic fungal infections, including *Aspergillus* spp. and *Pneumocystis jirovecii*, have long been a major threat to ICU patients, with IPA being the most common and fatal (3, 24). With the aging population, the widespread use of broad-spectrum antibiotics, antineoplastic agents, and immunosuppressants, and the increasing prevalence of organ transplantation, the proportion of immunocompromised patients in the ICU has been steadily rising (1, 2), leading to a yearly increase in the incidence of IPA (25).

The clinical symptoms of immunocompromised critically ill patients are often nonspecific, making the diagnosis of IPA challenging. Additionally, the increasing use of empirical antifungal therapy has led to a reduced pathogen detection rate in CMTs (26). Histopathology has traditionally been considered the gold standard for diagnosing IPA (15). However, obtaining a sufficient quantity of biopsy samples from the infection site can be challenging, leading to a high rate of false negatives (8). Additionally, lung biopsy is an invasive procedure, making it unsuitable for all patients. Fungal culture, microscopic smear, G and GM tests, BALF GM culture, and PCR are commonly used tools for diagnosing IPA. Unfortunately, these methods have limited diagnostic performance in immunocompromised patients due to factors, such as long turnaround times, low sensitivity, lack of standardized sampling, variability in mycological techniques, and the impact of prior antimicrobial treatment (9, 27, 28). Additionally, the nonspecific nature of radiological findings in pulmonary involvement significantly limits the diagnostic accuracy of chest CT in identifying IPA in these patients. Therefore, there is a need to explore new, efficient (faster and more accurate) diagnostic tools for IPA to enable early identification, timely adjustments to antibiotic regimens, and improved patient prognosis with reduced mortality.

mNGS, as a novel microbial diagnostic method, can accurately differentiate species and holds a significant value for the diagnosis of *Aspergillus* infection (7, 9, 11, 12, 29, 30). Shi Y et al. (7) and Zhan W et al. (31) have both demonstrated that mNGS exhibits good diagnostic performance in detecting IPA in immunocompromised patients. However, due to the lack of widely accepted standards and inherent technical limitations, the interpretation of mNGS results should still be approached with caution, particularly in immunocompetent patients (31). When the sequence reads of mNGS are relatively low, it can also be difficult to determine whether *Aspergillus* spp. is truly pathogenic or merely colonizing (32). Therefore, our study focuses on the diagnostic value of mNGS for IPA in immunocompromised critically ill patients, as well as exploring its potential clinical impact on this patient population.

In this study, we found that mNGS has a similar sensitivity (94.03% vs. 95.52%) and higher specificity (96.20% vs. 78.85%) and AUC (0.951 vs. 0.872) in diagnosing IPA compared to CMTs. Furthermore, mNGS was significantly superior to other single methods (Fig. 6B). mNGS only presented false positives in the diagnosis of four non-IPA patients (Fig. 1). This may be attributed to multiple factors, including the following: contamination of the microbial genome by environmental or endogenous microbiota and a high host genomic background with low microbial biomass of the true pathogen (33). After considering the negative CMT results and clinical symptoms, the final diagnosis was determined to be *Aspergillus* colonization. Among the 67 immunocompromised critically ill patients diagnosed with IPA, 28 (41.79%) were neutropenic, while 39 (58.21%) were non-neutropenia. This trend is consistent with the study by Bao S et al. (34), who reported that the number of non-neutropenia IPA patients is increasing year by year.

The Kappa analysis results indicated the agreement between the results of mNGS and CMTs (Kappa value = 0.638, *P* = 0.000). This suggests that mNGS and CMTs exhibit a high degree of diagnostic concordance, indicating that mNGS, similar to CMTs, demonstrates comparable diagnostic accuracy in identifying *Aspergillus* infections (Table 2). Further analysis revealed that when both mNGS and CMT results were positive in the IPA group, the proportion of completely matched pathogens was lower, while the probability of partial matches was higher (Fig. 5). This may be associated with the significantly higher rate of mixed infections in the IPA group (Fig. 3). The results in Fig. 3 show that the rate of mixed infections detected by mNGS was significantly higher than that of CMTs in both groups (*P* < 0.05), with the IPA group having a significantly higher proportion of mixed infections than the non-IPA group (*P* < 0.05). It is noteworthy that mNGS was able to diagnose 59 out of 60 cases of mixed infections, while CMTs could only diagnose 22 cases (Fig. 3). Among the 59 patients diagnosed with mixed infections by mNGS, the most common co-pathogens included bacteria, such as *Corynebacterium* spp. and MTB, fungi, such as *Candida* spp. and *Pneumocystis jirovecii*, and viruses, including EBV and CMV. This result is similar to the findings of Shi Y et al. (7), who reported that mNGS was able to diagnose 35 out of 37 cases with mixed infections, with *Pneumocystis jirovecii* and CMV being the most common co-pathogens. These findings suggest that mNGS has good diagnostic value for mixed infections compared to CMTs and indicate that the IPA group may be more susceptible to infections caused by mixed pathogens.

To further assess the impact of mNGS and CMT results on IPA patients, we classified the clinical effects of both methods into positive, negative, or no effect and presented the results using a Sankey diagram (Fig. 6C and D). The results showed that the positive effect of antibiotic regimen adjustments based on mNGS results was significantly higher than that of CMTs (90.70% vs 50%, *P* < 0.05), while negative and no effects were also lower. Among the 28-day prognosis influenced by clinical impact, mNGS results notably improved the prognosis in IPA patients compared to CMTs (Fig. 6C and D). This encouraging result suggests that mNGS results can guide antibiotic regimen adjustments in IPA patients and significantly enhance their prognosis.

### Limitation

First, the potential variability in sample collection, processing, and handling across different centers could impact the accuracy and consistency of mNGS and conventional microbiological test results. Additionally, as this study is a retrospective analysis, the use of histopathological results as the gold standard for confirming *Aspergillus* infection was not routinely applied, which may have influenced the findings. In future research, we will focus on increasing sample size, optimizing mNGS protocols, integrating mNGS with other diagnostic methods, and considering the inclusion of histopathological testing in prospective studies to improve IPA diagnosis.

We also recognize that the infrastructure and bioinformatics capacity for mNGS analysis may pose challenges for some laboratories. Smaller hospitals or institutions could consider collaborating with specialized facilities or outsourcing the analysis to commercial service providers.

### Conclusion

In summary, mNGS can serve as a highly sensitive method for detecting *Aspergillus* infections in immunocompromised critically ill patients, making it a valuable adjunct to CMTs. Moreover, mNGS accurately identifies mixed infections, aiding in appropriate antimicrobial regimen adjustments and ultimately improving the prognosis of these patients.

## Data Availability

The raw sequence data for this study have been uploaded and deposited in NCBI under BioProject no. PRJNA1264467. More deidentified data are available from corresponding author Jiancheng Zhuge (2456958062@qq.com) upon reasonable request.

## References

[1] Azoulay E, Pickkers P, Soares M, Perner A, Rello J, Bauer PR, van de Louw A, Hemelaar P, Lemiale V, Taccone FS, et al.. 2017. Acute hypoxemic respiratory failure in immunocompromised patients: the Efraim multinational prospective cohort study. Intensive Care Med 43:1808–1819. doi:10.1007/s00134-017-4947-1 28948369

[2] Kreitmann L, Helms J, Martin-Loeches I, Salluh J, Poulakou G, Pène F, Nseir S. 2024. ICU-acquired infections in immunocompromised patients. Intensive Care Med 50:332–349. doi:10.1007/s00134-023-07295-2 38197931

[3] Schauwvlieghe A, Rijnders BJA, Philips N, Verwijs R, Vanderbeke L, Van Tienen C, Lagrou K, Verweij PE, Van de Veerdonk FL, Gommers D, Spronk P, Bergmans D, Hoedemaekers A, Andrinopoulou E-R, van den Berg C, Juffermans NP, Hodiamont CJ, Vonk AG, Depuydt P, Boelens J, Wauters J, Dutch-Belgian Mycosis study group. 2018. Invasive aspergillosis in patients admitted to the intensive care unit with severe influenza: a retrospective cohort study. Lancet Respir Med 6:782–792. doi:10.1016/S2213-2600(18)30274-1 30076119

[4] Crum-Cianflone NF. 2016. Invasive aspergillosis associated with severe influenza infections. Open Forum Infect Dis 3:ofw171. doi:10.1093/ofid/ofw171 27704024 PMC5047415

[5] Patterson TF, Thompson GR 3rd, Denning DW, Fishman JA, Hadley S, Herbrecht R, Kontoyiannis DP, Marr KA, Morrison VA, Nguyen MH, Segal BH, Steinbach WJ, Stevens DA, Walsh TJ, Wingard JR, Young J-A, Bennett JE. 2016. Practice guidelines for the diagnosis and management of aspergillosis: 2016 update by the infectious diseases society of America. Clin Infect Dis 63:e1–e60. doi:10.1093/cid/ciw326 27365388 PMC4967602

[6] Cornillet A, Camus C, Nimubona S, Gandemer V, Tattevin P, Belleguic C, Chevrier S, Meunier C, Lebert C, Aupée M, Caulet-Maugendre S, Faucheux M, Lelong B, Leray E, Guiguen C, Gangneux J-P. 2006. Comparison of epidemiological, clinical, and biological features of invasive aspergillosis in neutropenic and nonneutropenic patients: a 6-year survey. Clin Infect Dis 43:577–584. doi:10.1086/505870 16886149

[7] Shi Y, Peng J-M, Hu X-Y, Yang Q-W, Wang Y. 2023. Metagenomic next-generation sequencing for detecting Aspergillosis pneumonia in immunocompromised patients: a retrospective study. Front Cell Infect Microbiol 13:1209724. doi:10.3389/fcimb.2023.1209724 38188627 PMC10770824

[8] Bialek R, Ernst F, Dietz K, Najvar LK, Knobloch J, Graybill JR, Schaumburg-Lever G. 2002. Comparison of staining methods and a nested PCR assay to detect Histoplasma capsulatum in tissue sections. Am J Clin Pathol 117:597–603. doi:10.1309/MH5B-GAQ2-KY19-FT7P 11939735

[9] Lin S, Chen Y, Li H, Chen T, Lin Q. 2024. Diagnostic value and clinical use of metagenomic next-generation sequencing for invasive pulmonary aspergillosis. Am J Transl Res 16:4885–4893. doi:10.62347/LDHU7380 39398609 PMC11470307

[10] Cordonnier C, Botterel F, Ben Amor R, Pautas C, Maury S, Kuentz M, Hicheri Y, Bastuji-Garin S, Bretagne S. 2009. Correlation between galactomannan antigen levels in serum and neutrophil counts in haematological patients with invasive aspergillosis. Clin Microbiol Infect 15:81–86. doi:10.1111/j.1469-0691.2008.02122.x 19154482

[11] Cai X, Sun C, Zhong H, Cai Y, Cao M, Wang L, Sun W, Tao Y, Ma G, Huang B, Yan S, Zhong J, Wang J, Lu Y, Guan Y, Song M, Wang Y, Li Y, Su X. 2024. The value of metagenomic next-generation sequencing with different nucleic acid extracting methods of cell-free DNA or whole-cell DNA in the diagnosis of non-neutropenic pulmonary aspergillosis. Front Cell Infect Microbiol 14:1398190. doi:10.3389/fcimb.2024.1398190 39135636 PMC11317373

[12] Hu W, Li X, Guo W, Shangguan Y, Xia J, Feng X, Sheng C, Ji Z, Ding C, Xu K. 2024. The utility of real-time PCR, metagenomic next-generation sequencing, and culture in bronchoalveolar lavage fluid for diagnosis of pulmonary aspergillosis. J Mol Diagn 26:832–842. doi:10.1016/j.jmoldx.2024.06.003 38972592

[13] Peng J-M, Du B, Qin H-Y, Wang Q, Shi Y. 2021. Metagenomic next-generation sequencing for the diagnosis of suspected pneumonia in immunocompromised patients. J Infect 82:22–27. doi:10.1016/j.jinf.2021.01.029 33609588

[14] Zhao J, Sun Y, Tang J, Guo K, Wang K, Zhuge J, Fang H. 2024. The clinical application of metagenomic next-generation sequencing in immunocompromised patients with severe respiratory infections in the ICU. Respir Res 25:360. doi:10.1186/s12931-024-02991-z 39369191 PMC11453054

[15] Donnelly JP, Chen SC, Kauffman CA, Steinbach WJ, Baddley JW, Verweij PE, Clancy CJ, Wingard JR, Lockhart SR, Groll AH, et al.. 2020. Revision and update of the consensus definitions of invasive fungal disease from the european organization for research and treatment of cancer and the mycoses study group education and research consortium. Clin Infect Dis 71:1367–1376. doi:10.1093/cid/ciz1008 31802125 PMC7486838

[16] Bolger AM, Lohse M, Usadel B. 2014. Trimmomatic: a flexible trimmer for Illumina sequence data. Bioinformatics 30:2114–2120. doi:10.1093/bioinformatics/btu170 24695404 PMC4103590

[17] Langmead B, Salzberg SL. 2012. Fast gapped-read alignment with Bowtie 2. Nat Methods 9:357–359. doi:10.1038/nmeth.1923 22388286 PMC3322381

[18] Wood DE, Lu J, Langmead B. 2019. Improved metagenomic analysis with Kraken 2. Genome Biol 20:257. doi:10.1186/s13059-019-1891-0 31779668 PMC6883579

[19] Miller S, Naccache SN, Samayoa E, Messacar K, Arevalo S, Federman S, Stryke D, Pham E, Fung B, Bolosky WJ, Ingebrigtsen D, Lorizio W, Paff SM, Leake JA, Pesano R, DeBiasi R, Dominguez S, Chiu CY. 2019. Laboratory validation of a clinical metagenomic sequencing assay for pathogen detection in cerebrospinal fluid. Genome Res 29:831–842. doi:10.1101/gr.238170.118B2 30992304 PMC6499319

[20] Chen H, Zheng Y, Zhang X, Liu S, Yin Y, Guo Y, Wang X, Zhang Y, Zhao C, Gai W, Wang H. 2024. Clinical evaluation of cell-free and cellular metagenomic next-generation sequencing of infected body fluids. J Adv Res 55:119–129. doi:10.1016/j.jare.2023.02.018 36889461 PMC10770109

[21] Wang J, Xu H, Wang X, Lan J. 2023. Rapid diagnosis of non-tuberculous mycobacterial pulmonary diseases by metagenomic next-generation sequencing in non-referral hospitals. Front Cell Infect Microbiol 12:1083497. doi:10.3389/fcimb.2022.1083497 36760234 PMC9902348

[22] Chen H, Yin Y, Gao H, Guo Y, Dong Z, Wang X, Zhang Y, Yang S, Peng Q, Liu Y, Wang H. 2020. Clinical utility of in-house metagenomic next-generation sequencing for the diagnosis of lower respiratory tract infections and analysis of the host immune response. Clin Infect Dis 71:S416–S426. doi:10.1093/cid/ciaa1516 33367583

[23] Yin G, Yin Y, Guo Y, Sun L, Ma S, Chen H, Wang Q, Wang H. 2025. Clinical impact of plasma metagenomic next-generation sequencing on infection diagnosis and antimicrobial therapy in immunocompromised patients. J Infect Dis 231:344–354. doi:10.1093/infdis/jiae343 39008608

[24] Azoulay E, Russell L, Van de Louw A, Metaxa V, Bauer P, Povoa P, Montero JG, Loeches IM, Mehta S, Puxty K, Schellongowski P, Rello J, Mokart D, Lemiale V, Mirouse A, the Nine-i Investigators. 2020. Diagnosis of severe respiratory infections in immunocompromised patients. Intensive Care Med 46:298–314. doi:10.1007/s00134-019-05906-5 32034433 PMC7080052

[25] Cabrera NL, George IA, Rauseo AM, Mazi P, Spec A. 2022. Novel agents in the treatment of invasive fungal infections in solid organ transplant recipients. Curr Opin Organ Transplant 27:235–242. doi:10.1097/MOT.0000000000000995 36354248

[26] Li N, Cai Q, Miao Q, Song Z, Fang Y, Hu B. 2021. High‐throughput metagenomics for identification of pathogens in the clinical settings. Small Methods 5:2000792. doi:10.1002/smtd.202000792 33614906 PMC7883231

[27] Hage CA, Carmona EM, Epelbaum O, Evans SE, Gabe LM, Haydour Q, Knox KS, Kolls JK, Murad MH, Wengenack NL, Limper AH. 2019. Microbiological laboratory testing in the diagnosis of fungal infections in pulmonary and critical care practice. An official American thoracic society clinical practice guideline. Am J Respir Crit Care Med 200:535–550. doi:10.1164/rccm.201906-1185ST 31469325 PMC6727169

[28] Arvanitis M, Mylonakis E. 2015. Diagnosis of invasive aspergillosis: recent developments and ongoing challenges. Eur J Clin Investigation 45:646–652. doi:10.1111/eci.12448 25851301

[29] Niu S, Liu D, Yang Y, Zhao L. 2024. Clinical utility of metagenomic next-generation sequencing in the diagnosis of invasive pulmonary aspergillosis in acute exacerbation of chronic obstructive pulmonary disease patients in the intensive care unit. Front Cell Infect Microbiol 14:1397733. doi:10.3389/fcimb.2024.1397733 39071167 PMC11272591

[30] Jiang Z, Gai W, Zhang X, Zheng Y, Jin X, Han Z, Ao G, He J, Shu D, Liu X, Zhou Y, Hua Z. 2024. Clinical performance of metagenomic next-generation sequencing for diagnosis of pulmonary Aspergillus infection and colonization. Front Cell Infect Microbiol 14:1345706. doi:10.3389/fcimb.2024.1345706 38606292 PMC11007027

[31] Zhan W, Liu Q, Yang C, Zhao Z, Yang L, Wang Y, Feng J. 2023. Evaluation of metagenomic next-generation sequencing diagnosis for invasive pulmonary aspergillosis in immunocompromised and immunocompetent patients. Mycoses 66:331–337. doi:10.1111/myc.13557 36541064

[32] Jia H, Liu H, Tu M, Wang Y, Wang X, Li J, Zhang G. 2023. Diagnostic efficacy of metagenomic next generation sequencing in bronchoalveolar lavage fluid for proven invasive pulmonary aspergillosis. Front Cell Infect Microbiol 13:1223576. doi:10.3389/fcimb.2023.1223576 37692168 PMC10484620

[33] Chen X, Ding S, Lei C, Qin J, Guo T, Yang D, Yang M, Qing J, He W, Song M, Zhang Y, Zeng H, Qin Q, Yang L, Long Y, Chen Y, Ma B, Ouyang R, Chen P, Luo H. 2020. Blood and bronchoalveolar lavage fluid metagenomic next-generation sequencing in pneumonia. Can J Infect Dis Med Microbiol 2020:6839103. doi:10.1155/2020/6839103 32879643 PMC7448216

[34] Bao S, Song H, Chen Y, Zhong C, Tang H. 2022. Metagenomic next-generation sequencing for the diagnosis of pulmonary aspergillosis in non-neutropenic patients: a retrospective study. Front Cell Infect Microbiol 12:925982. doi:10.3389/fcimb.2022.925982 35979088 PMC9376315

